# AMYTISS: Parallelized Automated Controller Synthesis for Large-Scale Stochastic Systems

**DOI:** 10.1007/978-3-030-53291-8_24

**Published:** 2020-06-16

**Authors:** Abolfazl Lavaei, Mahmoud Khaled, Sadegh Soudjani, Majid Zamani

**Affiliations:** 8 Microsoft Research Lab, Redmond, WA USA; 9 University of Southern California, Los Angeles, CA USA; 10 Department of Computer Science, LMU Munich, Munich, Germany; 11 Department of Electrical Engineering, TU Munich, Munich, Germany; 12 School of Computing, Newcastle University, Newcastle upon Tyne, UK; 13 Department of Computer Science, University of Colorado Boulder, Boulder, USA

**Keywords:** Parallel algorithms, Finite MDPs, Automated controller synthesis, Discrete-time stochastic systems, High performance computing platform

## Abstract

In this paper, we propose a software tool, called AMYTISS, implemented in C++/OpenCL, for designing correct-by-construction controllers for large-scale discrete-time stochastic systems. This tool is employed to (i) build finite Markov decision processes (MDPs) as finite abstractions of given original systems, and (ii) synthesize controllers for the constructed finite MDPs satisfying bounded-time high-level properties including safety, reachability and reach-avoid specifications. In AMYTISS, scalable parallel algorithms are designed such that they support the parallel execution within CPUs, GPUs and hardware accelerators (HWAs). Unlike all existing tools for stochastic systems, AMYTISS can utilize high-performance computing (HPC) platforms and cloud-computing services to mitigate the effects of the state-explosion problem, which is always present in analyzing large-scale stochastic systems. We benchmark AMYTISS against the most recent tools in the literature using several physical case studies including robot examples, room temperature and road traffic networks. We also apply our algorithms to a 3-dimensional autonomous vehicle and 7-dimensional nonlinear model of a BMW 320i car by synthesizing an autonomous parking controller.

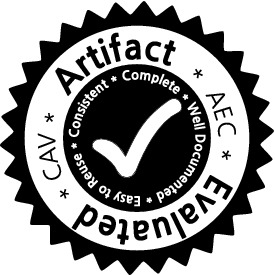



## Introduction

### Motivations

Large-scale stochastic systems are an important modeling framework to describe many real-life safety-critical systems such as power grids, traffic networks, self-driving cars, and many other applications. For this type of complex systems, automating the controller synthesis procedure to achieve high-level specifications, *e.g.,* those expressed as linear temporal logic (LTL) formulae [24], is inherently very challenging mainly due to their computational complexity arising from uncountable sets of states and actions. To mitigate the encountered difficulty, finite abstractions, *i.e.,* systems with finite state sets, are usually employed as replacements of original continuous-space systems in the controller synthesis procedure. More precisely, one can first abstract a given continuous-space system by a simpler one, *e.g.,* a finite Markov decision process (MDP), and then perform analysis and synthesis over the abstract model (using algorithmic techniques from computer science [3]). Finally, the results are carried back to the original system, while providing a guaranteed error bound [5, 13–21, 23].

Unfortunately, construction of finite MDPs for large-scale complex systems suffers severely from the so-called *curse of dimensionality*: the computational complexity grows exponentially as the number of state variables increases. To alleviate this issue, one promising solution is to employ high-performance computing (HPC) platforms together with cloud-computing services to mitigate the state-explosion problem. In particular, HPC platforms have a large number of processing elements (PEs) and this significantly affects the time complexity when serial algorithms are parallelized [7].

### Contributions

The main contributions and merits of this work are:

1. We propose a novel data-parallel algorithm for constructing finite MDPs from discrete-time stochastic systems and storing them in efficient distributed data containers. The proposed algorithm handles large-scale systems.
2. We propose a parallel algorithm for synthesizing discrete controllers using the constructed MDPs to satisfy safety, reachability, or reach-avoid specifications. More specifically, we introduce a parallel algorithm for the iterative computation of Bellman equation in standard dynamic programming [26, 27].
3. Unlike the existing tools in the literature, AMYTISS accepts bounded disturbances and natively supports both additive and multiplicative noises with different practical distributions including normal, uniform, exponential, and beta.

We apply the proposed implementations to real-world applications including robot examples, room temperature and road traffic networks, and autonomous vehicles. This extends the applicability of formal methods to some safety-critical real-world applications with high dimensions. The results show remarkable reductions in the memory usage and computation time outperforming all existing tools in the literature.

We provide AMYTISS as an *open-source* tool. After compilation, AMYTISS is loaded via pFaces [10] and launched for parallel execution within available parallel computing resources. The source of AMYTISS and detailed instructions on its building and running can be found in: https://github.com/mkhaled87/pFaces-AMYTISS

Due to lack of space, we provide details of traditional serial and proposed parallel algorithms, case studies, etc. in an arXiv version of the paper [12].

### Related Literature

There exist several software tools on verification and synthesis of stochastic systems with different classes of models. SReachTools [30] performs stochastic reachability analysis for linear, potentially time-varying, discrete-time stochastic systems. ProbReach [25] is a tool for verifying the probabilistic reachability for stochastic hybrid systems. SReach [31] solves probabilistic bounded reachability problems for two classes of models: (i) nonlinear hybrid automata with parametric uncertainty, and (ii) probabilistic hybrid automata with additional randomness for both transition probabilities and variable resets. Modest Toolset [6] performs modeling and analysis for hybrid, real-time, distributed and stochastic systems. Two competitions on tools for formal verification and policy synthesis of stochastic models are organized with reports in [1, 2].

FAUST$^{\mathsf 2}$ [29] generates formal abstractions for continuous-space discrete-time stochastic processes, and performs verification and synthesis for safety and reachability specifications. However, FAUST$^{\mathsf 2}$ is originally implemented in MATLAB and suffers from the curse of dimensionality due to its lack of scalability for large-scale models. StocHy [4] provides the quantitative analysis of discrete-time stochastic hybrid systems such that it constructs finite abstractions, and performs verification and synthesis for safety and reachability specifications.

AMYTISS differs from FAUST$^{\mathsf 2}$ and StocHy in two main directions. First, AMYTISS implements novel parallel algorithms and data structures targeting HPC platforms to reduce the undesirable effects of the state-explosion problem. Accordingly, it is able to perform parallel execution in different heterogeneous computing platforms including CPUs, GPUs and HWAs. Whereas, FAUST$^{\mathsf 2}$ and StocHy can only run serially on one CPU, and consequently, it is limited to small systems. Additionally, AMYTISS can handle the abstraction construction and controller synthesis for two and a half player games (*e.g.,* stochastic systems with bounded disturbances), whereas FAUST$^{\mathsf 2}$ and StocHy only handle one and a half player games (*e.g.,* disturbance-free systems).

Unlike all existing tools, AMYTISS offers highly scalable, distributed execution of parallel algorithms utilizing all available processing elements (PEs) in any heterogeneous computing platform. To the best of our knowledge, AMYTISS is the only tool of its kind for continuous-space stochastic systems that is able to utilize all types of compute units (CUs), simultaneously.

We compare AMYTISS with FAUST$^{\mathsf 2}$ and StocHy in Table 1 in detail in terms of different technical aspects. Although there have been some efforts in FAUST$^{\mathsf 2}$ and StocHy for parallel implementations, these are not compatible with HPC platforms. Specifically, FAUST$^{\mathsf 2}$ employs some parallelization techniques using parallel for-loops and sparse matrices inside Matlab, and StocHy uses Armadillo, a multi-threaded library for scientific computing. However, these tools are not designed for the parallel computation on HPC platforms. Consequently, they can only utilize CPUs and cannot run on GPUs or HWAs. In comparison, AMYTISS is developed in OpenCL, a language specially designed for data-parallel tasks, and supports heterogeneous computing platforms combining CPUs, GPUs and HWAs.

**Table 1.** Comparison between AMYTISS, FAUST$^{\mathsf 2}$ and StocHy based on native features.

Aspect	**FAUST**$^{\mathsf 2}$	**StocHy**	**AMYTISS**
Platform	CPU	CPU	All platforms
Algorithms	Serial on HPC	Serial on HPC	Parallel on HPC
Model	Stochastic control systems: linear, bilinear	Stochastic hybrid systems: linear, bilinear	Stochastic control systems: nonlinear
Specification	Safety, reachability	Safety, reachability	Safety, reachability, reach-avoid
Stochasticity	Additive noise	Additive noise	Additive & multiplicative noises
Distribution	Normal, user-defined	Normal, user-defined	Normal, uniform, exponential, beta, user-defined
Disturbance	Not supported	Not supported	Supported

Note that FAUST$^{\mathsf 2}$ and StocHy do not natively support reach-avoid specifications in the sense that users can explicitly provide some avoid sets. Implementing this type of properties requires some modifications inside those tools. In addition, we do not make a comparison here with SReachTools since it is mainly for stochastic reachability analysis of linear, potentially time-varying, discrete-time stochastic systems, while AMYTISS is not limited to reachability analysis and can handle nonlinear systems as well.

Note that we also provide a script in the tool repository^1^ that converts the MDPs constructed by AMYTISS into PRISM-input-files [11]. In particular, AMYTISS can natively construct finite MDPs from continuous-space stochastic control systems. PRISM can then be employed to perform the controller synthesis for those classes of complex specifications that AMYTISS does not support.

## Discrete-Time Stochastic Control Systems

We formally introduce discrete-time stochastic control systems (dt-SCS) below.

### Definition 1

A discrete-time stochastic control system (dt-SCS) is a tuple

1 $$\begin{aligned} \varSigma =\left( X,U,W,\varsigma ,f\right) , \end{aligned}$$

where,

- $X\!\subseteq \!\mathbb R^n$ is a Borel space as the state set and $(\!X, \!\mathcal B (X)\!)$ is its measurable space;
- $U\!\subseteq \! \mathbb R^m$ is a Borel space as the input set;
- $W\!\subseteq \! \mathbb R^p$ is a Borel space as the disturbance set;
- $\varsigma$ is a sequence of independent and identically distributed (i.i.d.) random variables from a sample space $\varOmega$ to a measurable set $\mathcal V_\varsigma$
  $$\begin{aligned} \varsigma :=\{\varsigma (k):\varOmega \rightarrow \mathcal V_{\varsigma },\,\,k\in {\mathbb {N}}\}; \end{aligned}$$
- $f:X\times U\times W\rightarrow X$ is a measurable function characterizing the state evolution of the system.

The state evolution of $\varSigma$, for a given initial state $x(0)\in X$, an input sequence $\nu (\cdot ):\mathbb N\rightarrow U$, and a disturbance sequence $w(\cdot ):\mathbb N\rightarrow W$, is characterized by the difference equations

2 $$\begin{aligned} \varSigma :x(k+1)=f(x(k),\nu (k),w(k)) + \varUpsilon (k), \quad \quad k\in \mathbb N, \end{aligned}$$

where $\varUpsilon (k) := \varsigma (k)$ with $\mathcal V_\varsigma = \mathbb R^n$ for the case of the additive noise, and $\varUpsilon (k) := \varsigma (k)x(k)$ with $\mathcal V_\varsigma$ equals to the set of diagonal matrices of the dimension *n* for the case of the multiplicative noise [22]. We keep the notation $\varSigma$ to indicate both cases and use respectively $\varSigma _\mathfrak a$ and $\varSigma _\mathfrak m$ when discussing these cases individually.

We should mention that our parallel algorithms are independent of the noise distribution. For an easier presentation of the contribution, we present our algorithms and case studies based on normal distributions but our tool natively supports other practical distributions including uniform, exponential, and beta. In addition, we provide a subroutine in our software tool so that the user can still employ the parallel algorithms by providing the density function of the desired class of distributions.

### Remark 1

Our synthesis is based on a $\max$-$\min$ optimization problem for two and a half player games by considering the disturbance and input of the system as players [9]. Particularly, we consider the disturbance affecting the system as an adversary and maximize the probability of satisfaction under the worst-case strategy of a rational adversary. Hence, we minimize the probability of satisfaction with respect to disturbances, and maximize it over control inputs.

One may be interested in analyzing dt-SCSs without disturbances (cf. case studies). In this case, the tuple (1) reduces to $\varSigma =(X,U,\varsigma ,f)$, where $f:X\times U\rightarrow X$, and the Eq. (2) can be re-written as

3 $$\begin{aligned} \varSigma :x(k+1)=f(x(k),\nu (k)) + \varUpsilon (k), \quad \quad k\in \mathbb N. \end{aligned}$$

Note that input models in this tool paper are given inside configuration text files. Systems are described by stochastic difference equations as (2)–(3), and the user should provide the right-hand-side of equations^2^. In the next section, we formally define MDPs and discuss how to build finite MDPs from given dt-SCSs.

## Finite Markov Decision Processes (MDPs)

A dt-SCS $\varSigma$ in (1) is *equivalently* represented by the following MDP [8, Proposition 7.6]:

$$\begin{aligned} \varSigma \!=\!\left( X,U,W,T_{\mathsf x}\right) \!, \end{aligned}$$

where the map $T_{\mathsf x}:\mathcal B(X)\times X\times U\times W\rightarrow [0,1]$, is a conditional stochastic kernel that assigns to any $x \in X$, $\nu \in U$, and $w\in W$, a probability measure $T_{\mathsf x}(\cdot | x,\nu , w)$. The alternative representation as the MDP is utilized in [28] to approximate a dt-SCS $\varSigma$ with a *finite* MDP $\widehat{\varSigma }$ using an abstraction algorithm. This algorithm first constructs a finite partition of the state set $X = \cup _i \mathsf X_i$, the input set $U = \cup _i \mathsf U_i$, and the disturbance set $W = \cup _i \mathsf W_i$. Then representative points $\bar{x}_i\in \mathsf X_i$, $\bar{\nu }_i\in \mathsf U_i$, and $\bar{w}_i\in \mathsf W_i$ are selected as abstract states, inputs, and disturbances. The transition probability matrix for the finite MDP $\widehat{\varSigma }$ is also computed as

4 $$\begin{aligned} \hat{T}_{\mathsf x} (x'|x,\nu ,w) = T_{\mathsf x} (\varXi (x')|x,\nu ,w),\quad \forall x, x'\in \hat{X}, \forall \nu \in \hat{U}, \forall w\in \hat{W}, \end{aligned}$$

where the map $\varXi :X\rightarrow 2^X$ assigns to any $x\in X$, the corresponding partition element it belongs to, *i.e.,* $\varXi (x) = \mathsf X_i$ if $x\in \mathsf X_i$. Since $\hat{X}$, $\hat{U}$ and $\hat{W}$ are finite sets, $\hat{T}_{\mathsf x}$ is a static map. It can be represented with a matrix and we refer to it, from now on, as the transition probability matrix.

For a given logic specification $\varphi$ and accuracy level $\epsilon$, the discretization parameter $\delta$ can be selected a priori such that

5 $$\begin{aligned} |\mathbb P(\varSigma \vDash \varphi ) - \mathbb P(\widehat{\varSigma }\vDash \varphi )|\le \epsilon , \end{aligned}$$

where $\epsilon$ depends on the horizon of formula $\varphi$, the Lipschitz constant of the stochastic kernel, and the *state* discretization parameter $\delta$ (cf. [28, Theorem 9]). We refer the interested reader to the arXiv version [12] for more details.

In the next sections, we propose novel parallel algorithms for the construction of finite MDPs and the synthesis of their controllers.

## Parallel Construction of Finite MDPs

In this section, we propose an approach to efficiently compute the transition probability matrix $\hat{T}_{\mathsf x}$ of the finite MDP $\widehat{\varSigma }$, which is essential for any controller synthesis procedure, as we discuss later in Sect. 5.

### Data-Parallel Threads for Computing $\hat{T}_{\mathsf X}$

The serial algorithm for computing $\hat{T}_{\mathsf x}$ is presented in Algorithm 1 in the arXiv version [12]. Computations of mean $\mu = f(\bar{x}_i,\bar{\nu }_j,\bar{w}_k, 0)$, $\text {PDF}(x\,|\,\mu , \mathsf {\varSigma })$, where PDF stands for probability density functions and $\mathsf {\varSigma }$ is a noise covariance matrix, and of $\hat{T}_{\mathsf x}$ all do not share data from one inner-loop to another. Hence, this is an embarrassingly data-parallel section of the algorithm. pFaces [10] can be utilized to launch necessary number of parallel threads on the employed hardware configuration (HWC) to improve the computation time of the algorithm. Each thread will eventually compute and store, independently, its corresponding values within $\hat{T}_{\mathsf x}$.

### Less Memory for Post States in $\hat{T}_{\mathsf X}$

$\hat{T}_{\mathsf x}$ is a matrix with the dimension of $(n_x\times n_\nu \times n_w,n_x)$. The number of columns is $n_x$ as we need to compute and store the probability for each reachable partition element $\varXi (x'_l)$, corresponding to the representing post state $x'_l$. Here, we consider the Gaussian PDFs for the sake of a simpler presentation. For simplicity, we now focus on the computation of tuple $(\bar{x}_i, \bar{\nu }_j, \bar{w}_k)$. In many cases, when the PDF is decaying fast, only partition elements near $\mu$ have high probabilities of being reached, starting from $\bar{x}_i$ and applying an input $\bar{\nu }_j$.

We set a cutting probability threshold $\gamma \in [0,1]$ to control how many partition elements around $\mu$ should be stored. For a given mean value $\mu$, a covariance matrix $\mathsf {\varSigma }$ and a cutting probability threshold $\gamma$, $x \in X$ is called a PDF cutting point if $\gamma = \text {PDF}(x \vert \mu , \mathsf {\varSigma })$. Since Gaussian PDFs are symmetric, by repeating this cutting process dimension-wise, we end up with a set of points forming a hyper-rectangle in *X*, which we call it the cutting region and denote it by $\hat{X}^{\mathsf {\varSigma }}_{\gamma }$. This is visualized in Fig. 1 in the arXiv version [12] for a 2-dimensional system. Any partition element $\varXi (x'_l)$ with $x'_l$ outside the cutting region is considered to have zero probability of being reached. Such approximation allows controlling the sparsity of the columns of $\hat{T}_{\mathsf x}$. The closer the value of $\gamma$ to zero, the more accurate $\hat{T}_{\mathsf x}$ in representing transitions of $\widehat{\varSigma }$. On the other hand, the closer the value of $\gamma$ to one, less post state values need to be stored as columns in $\hat{T}_{\mathsf x}$. The number of probabilities to be stored for each $(\bar{x}_i, \bar{\nu }_j, \bar{w}_k)$ is then $\vert \hat{X}^{\mathsf {\varSigma }}_{\gamma } \vert$.

Note that since $\mathsf {\varSigma }$ is fixed prior to running the algorithm, number of columns needed for a fixed $\gamma$ can be identified before launching the computation. We can then accurately allocate a uniform fixed number of memory locations for any tuple $(\bar{x}_i, \bar{\nu }_j, \bar{w}_k)$ in $\hat{T}_{\mathsf x}$. Hence, there is no need for a dynamic sparse matrix data structure and $\hat{T}_{\mathsf x}$ is now a matrix with a dimension of $(n_x\times n_\nu \times n_w,\vert \hat{X}^{\mathsf {\varSigma }}_{\gamma } \vert )$.

### A Parallel Algorithm for Constructing Finite MDP $\widehat{\varSigma }$

We present a novel parallel algorithm (Algorithm 2 in the arXiv version [12]) to efficiently construct and store $\hat{T}_{\mathsf x}$ as a successor. We employ the discussed enhancements in Subsect. 4.1 and 4.2 within the proposed algorithm. We do not parallelize the for-loop in Algorithm 2, Step 2, to avoid excessive parallelism (*i.e.,* we parallelize loops only over *X* and *U*, but not over *W*). Note that, practically, for large-scale systems, $\vert \hat{X} \times \hat{U} \vert$ can reach up to billions. We are interested in the number of parallel threads that can be scheduled reasonably by available HW computing units.

## Parallel Synthesis of Controllers

In this section, we employ dynamic programming to synthesize controllers for constructed finite MDPs $\widehat{\varSigma }$ satisfying safety, reachability, and reach-avoid properties [26, 27]. The classical serial algorithm and its proposed parallelized version are respectively presented as Algorithms 3 and 4 in the arXiv version [12]. We should highlight that the parallelism here mainly comes from the parallelization of matrix multiplication and the loop over time-steps cannot be parallelized due to the data dependency. More details can be found in the arXiv version.

### On-the-Fly Construction of $\hat{T}_{\mathsf X}$

In AMYTISS, we also use another technique that further reduces the required memory for computing $\hat{T}_{\mathsf x}$. We refer to this approach as *on-the-fly abstractions* (OFA). In OFA version of Algorithm 4 [12], we skip computing and storing the MDP $\hat{T}_{\mathsf x}$ and the matrix $\hat{T}_{0\mathsf x}$ (*i.e.,* Steps 1 and 5). We instead compute the required entries of $\hat{T}_{\mathsf x}$ and $\hat{T}_{0\mathsf x}$ on-the-fly as they are needed (*i.e.,* Steps 13 and 15). This significantly reduces the required memory for $\hat{T}_{\mathsf x}$ and $\hat{T}_{0\mathsf x}$ but at the cost of repeated computation of their entries in each time step from 1 to $T_d$. This gives the user an additional control over the trade-off between the computation time and memory.

### Supporting Multiplicative Noises and Practical Distributions

AMYTISS natively supports multiplicative noises and practical distributions such as uniform, exponential, and beta distributions. The technique introduced in Subsect. 4.2 for reducing the memory usage is also tuned for other distributions based on the support of their PDFs. Since AMYTISS is designed for extensibility, it allows also for customized distributions. Users need to specify their desired PDFs and hyper-rectangles enclosing their supports so that AMYTISS can include them in the parallel computation of $\hat{T}_{\mathsf x}$. Further details on specifying customized distributions are provided in the README file.

AMYTISS also supports multiplicative noises as introduced in (2). Currently, the memory reduction technique of Subsect. 4.2 is disabled for systems with multiplicative noises. This means users should expect larger memory requirements for systems with multiplicative noises. However, users can still benefit from the proposed OFA version to compensate for the increase in memory requirement. We plan to include this feature for multiplicative noises in a future update of AMYTISS. Note that for a better demonstration, previous sections were presented by the additive noise and Gaussian normal PDF to introduce the concepts.

## Benchmarking and Case Studies

AMYTISS is self-contained and requires only a modern C++ compiler. It supports all major operating systems: Windows, Linux and Mac OS. Once compiled, utilizing AMYTISS is a matter of providing text configuration files and launching the tool. AMYTISS implements scalable parallel algorithms that run on top of pFaces [10]. Hence, users can utilize computing power in HPC platforms and cloud computing to scale the computation and control the computational complexities of their problems. Table 2 lists the HW configuration we use to benchmark AMYTISS. The devices range from local devices in desktop computers to advanced compute devices in Amazon AWS cloud computing services.

**Table 2.** HW configurations for benchmarking AMYTISS.

Id	Description	PEs	Frequency
CPU$_1$	Local machine: Intel Xeon E5-1620	8	3.6 GHz
CPU$_2$	Macbook Pro 15: Intel i9-8950HK	12	2.9 GHz
CPU$_3$	AWS instance c5.18xlarge: Intel Xeon Platinum 8000	72	3.6 GHz
GPU$_1$	Macbook Pro 15 laptop laptop: Intel UHD Graphics 630	23	0.35 GHz
GPU$_2$	Macbook Pro 15 laptop: AMD Radeon Pro Vega 20	1280	1.2 GHz
GPU$_3$	AWS p3.2xlarge instance: NVIDIA Tesla V100	5120	0.8 GHz

Table 3 shows the benchmarking results running AMYTISS with these HWCs for several case studies and makes comparisons between AMYTISS, FAUST$^{\mathsf 2}$, and StocHy. We employ a machine with Windows operating system (Intel i7@3.6 GHz CPU and 16 GB of RAM) for FAUST$^{\mathsf 2}$, and StocHy. It should be mentioned that FAUST$^{\mathsf 2}$ predefines a minimum number of representative points based on the desired abstraction error, and accordingly the computation time and memory usage reported in Table 3 are based on the minimum number of representative points. In addition, to have a fair comparison, we run all the case studies with additive noises since neither FAUST$^{\mathsf 2}$ nor StocHy supports multiplicative noises.

To show the applicability of our results to large-scale systems, we apply our techniques to several physical case studies. We synthesize controllers for 3- and 5-dimensional *room temperature networks* to keep temperatures in a comfort zone. Furthermore, we synthesize controllers for *road traffic networks* with 3 and 5 dimensions to keep the density of the traffic below some desired level. In addition, we apply our algorithms to a 2-dimensional nonlinear robot and synthesize controllers satisfying safety and reach-avoid specifications. Finally, we consider 3- and 7-dimensional *nonlinear* models of an autonomous vehicle and synthesize reach-avoid controllers to automatically park the vehicles. For details of case studies, see the arXiv version [12].

Table 3 presents a comparison between AMYTISS, FAUST$^{\mathsf 2}$ and StocHy w.r.t the computation time and required memory. For each HWC, we show the time in seconds to solve the problem. Clearly, employing HWCs with more PEs reduces the time to solve the problem. This is a strong indication for the scalability of the proposed algorithms. Since AMYTISS is the only tool for stochastic systems that can utilize the reported HWCs, we do not compare it with other similar tools.

**Table 3.** Comparison between AMYTISS, FAUST$^{\mathsf 2}$ and StocHy based on their native features for several (physical) case studies. CSB refers to the continuous-space benchmark provided in [4]. $\dagger$ refers to cases when we run AMYTISS with the OFA algorithm. N/M refers to the situation when there is not enough memory to run the case study. N/S refers to the lack of native support for nonlinear systems. (Kx) refers to an 1000-times speedup. The presented speedup is the maximum speedup value across all reported devices. The required memory usage and computation time for FAUST$^{\mathsf 2}$ and StocHy are reported for just constructing finite MDPs. The reported times and memories are respectively in seconds and MB, unless other units are denoted.

Problem	Spec.	$\vert \hat{X} \times \hat{U} \vert$	$T_d$	**AMYTISS** (time)							**FAUST**$^{\mathsf 2}$		**fStocHy**		Speedup w.r.t	
				Mem.	CPU$_1$	CPU$_2$	CPU$_3$	GPU$_1$	GPU$_2$	GPU$_3$	Mem.	Time	Mem.	Time	FAUST	StocHy
2-d StocHy CSB	Safety	4	6	$\le$1.0	$\le$1.0	$\le$1.0	$\le$1.0	$\le$1.0	$\le$1.0	0.0001	$\le$1.0	0.002	8.5	0.015	**20 x**	**150 x**
3-d StocHy CSB	Safety	8	6	$\le$1.0	$\le$1.0	$\le$1.0	$\le$1.0	$\le$1.0	$\le$1.0	0.0001	$\le$1.0	0.002	8.5	0.08	**20 x**	**800 x**
4-d StocHy CSB	Safety	16	6	$\le$1.0	$\le$1.0	$\le$1.0	$\le$1.0	$\le$1.0	$\le$1.0	0.0002	$\le$1.0	0.01	8.5	0.17	**50 x**	**850 Kx**
5-d StocHy CSB	Safety	32	6	$\le$1.0	$\le$1.0	$\le$1.0	$\le$1.0	$\le$1.0	$\le$1.0	0.0003	$\le$1.0	0.01	8.7	0.54	**33 x**	**1.8 Kx**
6-d StocHy CSB	Safety	64	6	$\le$1.0	$\le$1.0	$\le$1.0	$\le$1.0	$\le$1.0	$\le$1.0	0.0006	4.251	1.2	9.6	2.17	**2.0 Kx**	**3.6 Kx**
7-d StocHy CSB	Safety	128	6	$\le$1.0	$\le$1.0	$\le$1.0	$\le$1.0	$\le$1.0	$\le$1.0	0.0012	38.26	6	12.9	9.57	**5 Kx**	**7.9 Kx**
8-d StocHy CSB	Safety	256	6	$\le$1.0	$\le$1.0	$\le$1.0	$\le$1.0	$\le$1.0	$\le$1.0	0.0026	344.3	37	26.6	40.5	**14.2 Kx**	**15.6 Kx**
9-d StocHy CSB	Safety	512	6	1.0	$\le$1.0	$\le$1.0	$\le$1.0	$\le$1.0	$\le$1.0	0.0057	3 GB	501	80.7	171.6	**87.8 Kx**	**30.1 Kx**
10-d StocHy CSB	Safety	1024	6	4.0	$\le$1.0	$\le$1.0	$\le$1.0	$\le$1.0	$\le$1.0	0.0122	N/M		297.5	385.5	N/A	**32 Kx**
11-d StocHy CSB	Safety	2048	6	16.0	1.0912	$\le$1.0	$\le$1.0	$\le$1.0	$\le$1.0	0.0284	N/M		1 GB	1708.2	N/A	**60 Kx**
12-d StocHy CSB	Safety	4096	6	64.0	4.3029	4.1969	$\le$1.0	$\le$1.0	$\le$1.0	0.0624	N/M		4 GB	11216	N/A	**179 Kx**
13-d StocHy CSB	Safety	8192	6	256.0	18.681	19.374	1.8515	1.6802	$\le$1.0	0.1277	N/M		N/A	$\ge$24 h	N/A	$\ge$**676 Kx**
14-d StocHy CSB	Safety	16384	6	1024.0	81.647	94.750	7.9987	7.3489	6.1632	0.2739	N/M		N/A	$\ge$24 h	N/A	$\ge$**320 Kx**
2-d Robot$\dagger$	Safety	203401	8	$\le$1.0	8.5299	5.0991	0.7572	$\le$1.0	$\le$1.0	0.0154	N/A		N/A		N/A	N/A
2-d Robot	R.Avoid	741321	16	482.16	48.593	18.554	4.5127	2.5311	3.4353	0.3083	N/S		N/S		N/A	N/A
2-d Robot$\dagger$	R.Avoid	741321	16	4.2484	132.10	41.865	11.745	5.3161	3.6264	0.1301	N/A		N/A		N/A	N/A
3-d Room Temp.	Safety	7776	8	6.4451	0.1072	0.0915	0.0120	$\le$1.0	$\le$1.0	0.0018	3.12	1247	N/M		**692 Kx**	N/A
3-d Room Temp.$\dagger$	Safety	7776	8	$\le$1.0	0.5701	0.3422	0.0627	$\le$1.0	$\le$1.0	0.0028	N/A		N/A		N/A	N/A
5-d Room Temp.	Safety	279936	8	3338.4	200.00	107.93	19.376	10.084	N/M	1.8663	2 GB	3248	N/M		**1740 x**	N/A
5-d Room Temp.$\dagger$	Safety	279936	8	1.36	716.84	358.23	63.758	30.131	22.334	0.5639	N/A		N/A		N/A	N/A
3-d Road Traffic	Safety	2125764	16	1765.7	29.200	131.30	3.0508	5.7345	10.234	1.2895	N/M		N/M		N/A	N/A
3-d Road Traffic$\dagger$	Safety	2125764	16	14.19	160.45	412.79	13.632	12.707	11.657	0.3062	N/A		N/A		N/A	N/A
5-d Road Traffic	Safety	68841472	7	8797.4	N/M	537.91	38.635	N/M	N/M	4.3935	N/M		N/M		N/A	N/A
5-d Road Traffic$\dagger$	Safety	68841472	7	393.9	1148.5	1525.1	95.767	44.285	36.487	0.7397	N/A		N/A		N/A	N/A
3-d Vehicle	R.Avoid	1528065	32	1614.7	2.5 h	1.1 h	871.89	898.38	271.41	10.235	N/S		N/S		N/A	N/A
3-d Vehicle$\dagger$	R.Avoid	1528065	32	11.17	2.8 h	1.9 h	879.78	903.2	613.55	107.68	N/A		N/A		N/A	N/A
7-d BMW 320i	R.Avoid	3937500	32	10169.4	N/M	$\ge$24 h	21.5 h	N/M	N/M	825.62	N/S		N/S		N/A	N/A
7-d BMW 320i$\dagger$	R.Avoid	3937500	32	30.64	$\ge$24 h	$\ge$24 h	$\ge$24 h	$\ge$24 h	$\ge$24 h	1251.7	N/A		N/A		N/A	N/A

In Table 3, first 13 rows, we also include the benchmark provided in StocHy [4, Case study 3]. Table 4 in the arXiv version [12] shows an additional comparison between StocHy and AMYTISS on a machine with the same configuration as the one employed in [4] (a laptop having an Intel Core i$7-8550$U CPU at 1.80GHz with 8 GB of RAM). StocHy suffers significantly from the state-explosion problem as seen from its exponentially growing computation time. AMYTISS, on the other hand, outperforms StocHy and can handle bigger systems using the same hardware.

As seen in Table 3, AMYTISS outperforms FAUST$^{\mathsf 2}$ and StocHy in all the case studies (maximum speedups up to 692000 times). Moreover, AMYTISS is the only tool that can utilize the available HW resources. The OFA feature in AMYTISS reduces dramatically the required memory, while still solves the problems in a reasonable time. FAUST$^{\mathsf 2}$ and StocHy fail to solve many of the problems since they lack the native support for nonlinear systems, they require large amounts of memory, or they do not finish computing within 24 hours.

Note that considering only dimensions of systems can be sometimes misleading. In fact, number of transitions in MDPs ($\vert \hat{X} \times \hat{U} \vert$) can give a better judgment on the size of systems since it directly affects the memory/time needed for solving the problem. For instance in Table 3, the number of transitions for the 14-dimensional case study is 16384, while for the 5-dimensional room temperature example is 279936 transitions (*i.e.,* almost 17 times bigger). This means AMYTISS can clearly handle much larger systems than existing tools.

## Footnotes

1 https://github.com/mkhaled87/pFaces-AMYTISS/blob/master/interface/export PrismMDP.m.

2 An example of such a configuration file is provided at: https://github.com/mkhaled 87/pFaces-AMYTISS/blob/master/examples/ex-toy-safety/toy2d.cfg.
